# Response to Azacytidine in a Patient With Refractory Peripheral T-cell Lymphoma With TET2 Mutation

**DOI:** 10.7759/cureus.65416

**Published:** 2024-07-26

**Authors:** Minh-Anh Le, Feras Al-Moussally, Allison Carilli

**Affiliations:** 1 Internal Medicine, University of Central Florida (UCF)HCA Florida Healthcare (Greater Orlando) Internal Medicine Residency Program, Orlando, USA; 2 Oncology, University of Central Florida College of Medicine, Orlando, USA

**Keywords:** azacytidine, azacitidine, t-cell lymphoma, peripheral t-cell lymphoma not otherwise specified, ptcl-nos, ptcl

## Abstract

Peripheral T-cell lymphomas (PTCLs) are an aggressive form of non-Hodgkin lymphomas. PTCLs have multiple subtypes, with PTCL not otherwise specified (PTCL-NOS) being the most common. This subtype usually has a high rate of relapse. Making an accurate diagnosis requires molecular genetic analyses, histopathological examination, and immunophenotyping. Treatment for PTCL traditionally starts with the CHOP regimen (cyclophosphamide, doxorubicin, vincristine, and prednisone). We present a case of a patient with PTCL-NOS who progressed despite multiple treatment regimens, including both traditional and novel therapeutic agents, and finally achieved good results with azacytidine, selected based on a *TET2 *mutation. This case proposes future research into Azacytidine’s efficacy in this patient population and further exploration of the broader utility of epigenetic therapies in PTCL.

## Introduction

Peripheral T-cell lymphomas (PTCLs) constitute about 5%-15% of all non-Hodgkin lymphomas [1,2]. They are a diverse group of rare and aggressive malignancies derived from mature T-cell lymphocytes. There are 27 different types of PTCL [3]. However, PTCL not otherwise specified (PTCL-NOS) remains the most common subtype. They account for roughly 30% of PTCL cases [4], highlighting the classification's broad and somewhat ambiguous nature. Diagnosis relies on a combination of histopathological examination, immunophenotyping, and molecular genetic analyses to identify specific gene rearrangements and mutations, such as *TET2*, which are implicated in the disease's pathogenesis through epigenetic mechanisms.

The treatment landscape for PTCL is evolving, with the CHOP regimen (cyclophosphamide, doxorubicin, vincristine, and prednisone) being a traditional first-line therapy [5]. However, outcomes for patients with relapsed or refractory PTCL remain dismal with conventional salvage regimens [6]. In this context, azacytidine has emerged as a promising agent, particularly in PTCL subtypes characterized by specific genetic mutations. Azacytidine’s mechanism of action involves the inhibition of DNA methylation, potentially restoring normal gene function in cancer cells. Recent research has begun to explore the efficacy of azacytidine, alone or in combination with other therapies, in improving responses in PTCL, especially in cases expressing T-follicular helper cell markers or harboring mutations like *TET2* [7]. This case report explores azacytidine’s effectiveness in a refractory PTCL-NOS patient, emphasizing the pivotal role of genetic and epigenetic insights in therapeutic decision-making.

## Case presentation

We present a case of a 67-year-old man with PTCL. He initially presented with painless enlarged lymph nodes in his neck and occasional night sweats for several months. The patient underwent right neck dissection at age 57, and pathology analysis revealed atypical lymphocytes, clonal T-cell gene rearrangements, and suspected atypical Reed-Sternberg cells but later classified as reactive on a second review.

**Figure 1:**
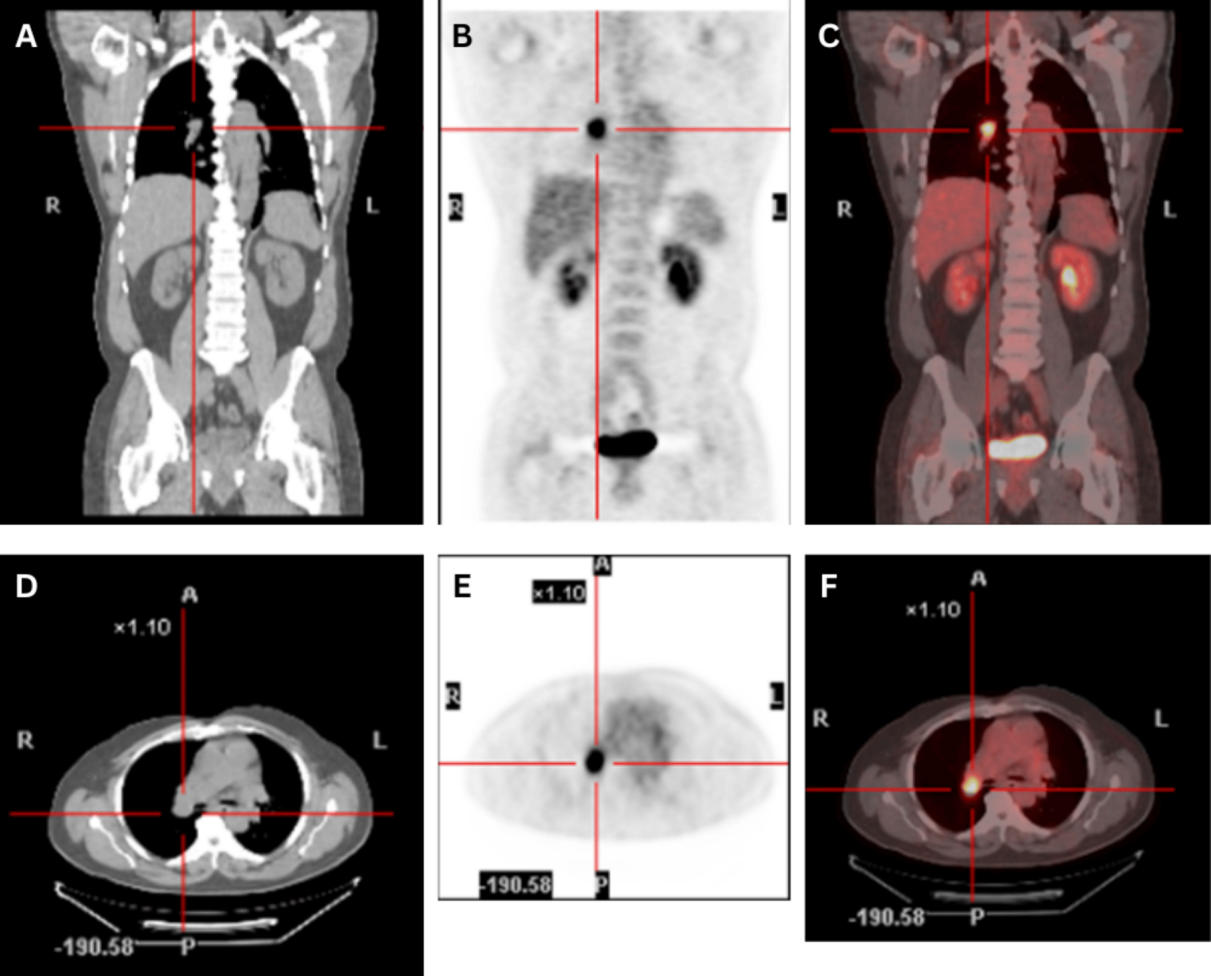
Intensely FDG-avid right hilar lymph node on PET/CT A: CT Coronal view. B: PET Coronal view. C: Fused Coronal view. D: CT Transaxial view. E: PET Transaxial view. F: Fused Transaxial view.

Subsequent surveillance PET scans over the following three years showed progressing lymphadenopathy with increased FDG avidity in multiple regions including the neck, chest, abdomen, and pelvis, again raising suspicion of lymphoma (Figures 1A-1F). A biopsy of a posterior cervical lymph node revealed non-necrotizing epithelioid granulomas and a monoclonal T-cell population. Clonal rearrangements of the T-cell receptor gamma (TCRG) and beta (TCRB) genes were detected by polymerase chain reaction (PCR). His case was sent to the National Institutes of Health (NIH) for review and the pathological diagnosis was determined to be PTCL-NOS.

The patient was initiated on chemotherapy with a CHOP regimen. Partial response was seen after three cycles and treatment was continued. However, after six cycles of CHOP, a repeat PET scan showed progressive disease. He was then treated with a salvage regimen of gemcitabine and oxaliplatin, achieving a complete metabolic response. Consolidative autologous stem cell transplant was performed as well.

**Figure 2:**
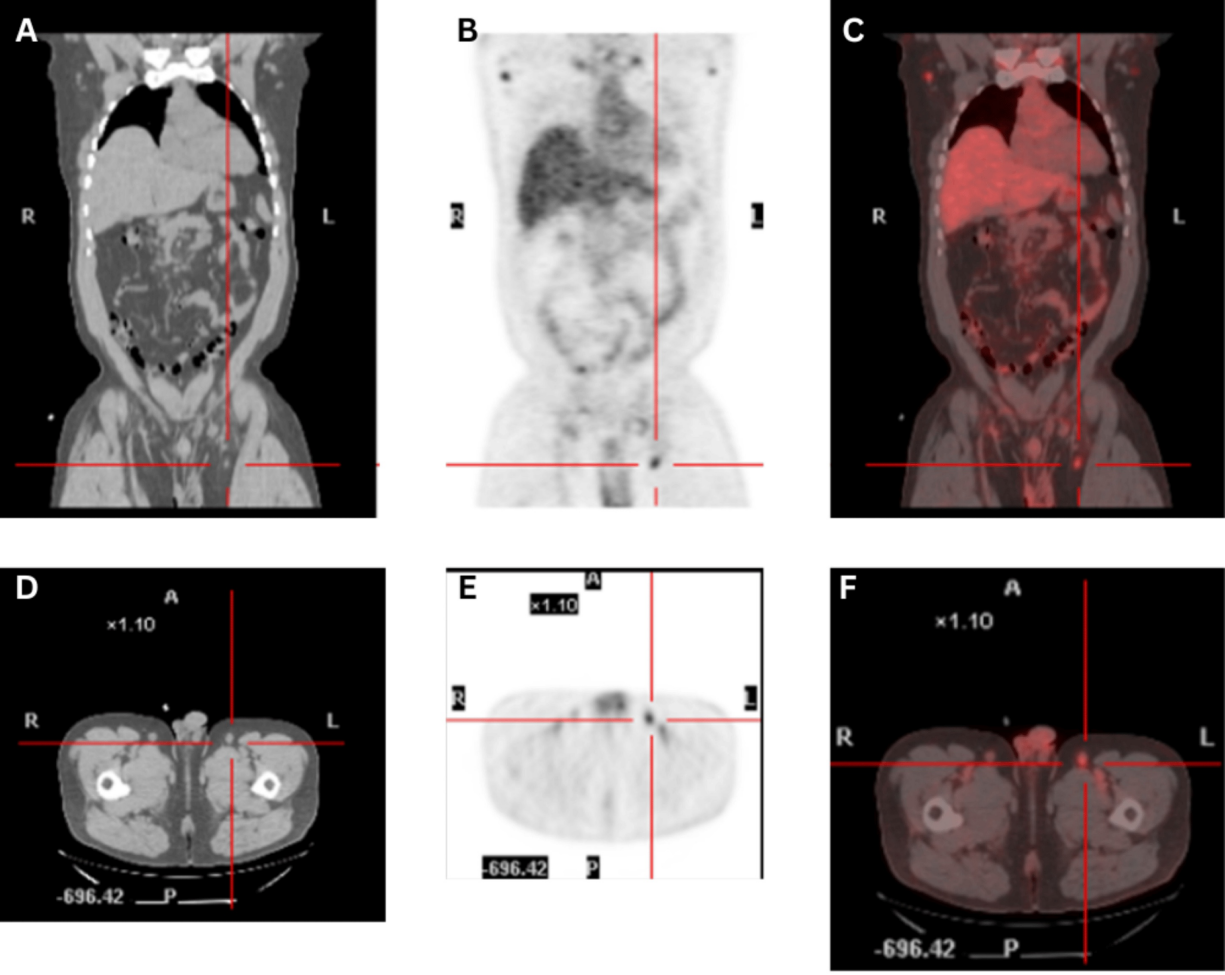
Intensely FDG-avid left inguinal lymph node on PET/CT (A) CT Coronal view. (B) PET Coronal view. (C) Fused Coronal view. (D) CT Transaxial view. (E) PET Transaxial view. (F) Fused Transaxial view.

Surveillance PET scan one year after transplant revealed new multilevel hypermetabolic nodes in the neck, thorax, abdomen, pelvis, or inguinal regions, consistent with recurrent T-cell lymphoma (Figures 2A-2F). A biopsy of the right inguinal lymph node revealed PTCL with lymphoepithelioid pattern or Lennert’s Lymphoma. The immunophenotypic features in this case are summarized in Table 1.

**Table 1:** Immunophenotypic features of the right inguinal lymph node biopsy

Marker	Expression
CD3	Positive
CD4	Positive
CD5	Positive
CD43	Positive
CD2	Positive
CD7	Positive
CD8	Positive
Beta F1	Positive in the majority of T-cells
CD21	Highlighted the broken dendritic meshwork of residual follicles
CD30	Positive in several large cells
CD15	Highlighted only the histiocytes

Salvage therapy with brentuximab and romidepsin was again rapidly outpaced by disease progression. Genetic testing from the bone marrow revealed a Ten-Eleven Translocation 2 (*TET2*) mutation as the only clinically significant genomic finding. Azacytidine was initiated following this result, and a partial response was achieved after five cycles. A PET scan showed only persistent FDG-avid cervical lymph nodes, with the previously noted FDG-avid lymph nodes in the neck, chest, abdomen, and pelvis no longer demonstrating metabolic activity. This was the best result obtained from all treatment courses, aside from the complete response achieved after autologous transplantation. At this time, the patient decided to go ahead with haploidentical stem cell transplantation from his daughter, understanding the risks and benefits of the procedure. He then finished six cycles of azacytidine consolidation with a follow-up PET scan showing a complete response. The patient is currently under observation and has remained in remission for five years now since completing adjuvant therapy with no complications.

## Discussion

PTCL-NOS exhibits a notably high rate of relapse and refractory disease. A prospective study has indicated that up to 68% of PTCL cases relapsed or were refractory following first-line treatment with the median overall survival for these patients being less than six months, highlighting the urgent need for more effective therapeutic strategies [8]. Traditional salvage therapies, borrowed from regimens for aggressive B-cell lymphomas, have yielded limited success and while novel agents like Belinostat, Brentuximab Vedotin, and Pralatrexate offer new options, the efficacy of these agents varies and significant survival benefits are rare. This hurdle highlights the need for personalized treatments tailored to the genetic makeup of the disease. Advances in novel agents, particularly histone deacetylase (HDAC) inhibitors, and strategies such as high-dose chemotherapy and stem cell transplantation seem promising in certain patients, emphasizing the importance of genetic profiling in treatment decisions. Allogeneic stem cell transplantation, while associated with significant transplant-related mortality, is also a potentially curative approach for eligible patients [9,10].

The *TET2* gene, implicated in epigenetic regulation via DNA methylation, is frequently mutated in angioimmunoblastic T-cell lymphoma (AITL) and to a lesser extent in PTCL-NOS. According to a review of previous studies, mutations in *TET2* have been identified in 47%-100% of AITL cases, a prevalence significantly higher than the 14.6%-38% observed in PTCL-NOS [11]. This differential mutation frequency suggests a distinct pathogenetic role of *TET2* across PTCL subtypes and may inform therapeutic targeting.

Hypomethylating agents, notably azacytidine, have been effective in addressing *TET2* mutations [12]. Their therapeutic use in AITL and PTCL-NOS with these mutations is based on their capacity to correct the abnormal methylation patterns caused by *TET2* alterations. Our review of the literature reveals encouraging results, with a significant number of AITL patients responding to azacytidine treatment, underscoring its potential as a viable treatment approach given the prevalent *TET2* mutation in AITL. Specifically, a retrospective study reported a 75% overall response rate (ORR) in 12 AITL patients treated with azacytidine for *TET2* mutations [13]. Furthermore, another study documented a complete response in three of four patients with *TET2*-mutated AITL treated with a combination of azacytidine and Venetoclax [14]. Another study involving 27 PTCL patients receiving a combination of azacytidine and romidepsin also yielded promising results [15]. A prospective study introducing azacytidine plus CHOP as an initial treatment for PTCL showed sustained remission in 75% of evaluable patients, with *TET2* mutations significantly associated with complete response, improved progression-free survival, and overall survival [16]. This evidence suggests that targeting aberrant DNA methylation in PTCL with *TET2* mutation could offer an alternative to conventional chemotherapy regimens, though this hypothesis needs to be studied further.

In our case, the patient's disease progressed despite multiple treatment regimens, including traditional chemotherapy, novel therapeutic agents, and an autologous transplant. This mirrors findings from prior studies that highlight the inconsistent effectiveness of such treatments in managing relapsed/refractory PTCL-NOS.

Given the patient's failure with other therapies and supported by genetic evidence, we initiated azacytidine after confirming a *TET2* mutation. The patient achieved a partial response following five cycles of azacytidine, leading to sustained complete remission after subsequent allogeneic hematopoietic stem cell transplantation. To our knowledge, no studies have reported on the response rate of single-agent azacytidine in patients with mutated *TET2* PTCL-NOS. This case underscores the need for further research into azacytidine’s effectiveness in a broader cohort of PTCL-NOS patients with *TET2* mutations.

It was noted that the patient achieved a partial response after azacytidine and a complete response following allogeneic hematopoietic stem cell transplantation. While this sequence of treatments made it difficult to determine the exact impact of azacytidine, we hypothesize that it played a crucial role, as the patient achieved the best response with its use compared to previous regimens and has remained disease-free for five years without relapse. One study reported five-year overall survival and progression-free survival rates of 58.9% and 52.6%, respectively, after allogeneic hematopoietic stem cell transplantation [17]. It also suggested that allogeneic transplantation benefits patients with high-risk disease who achieve at least partial remission before the procedure, while those with refractory or progressive disease do not benefit [17]. This further supports our use of azacytidine to achieve partial remission before proceeding with allogeneic hematopoietic stem cell transplantation with curative intent.

## Conclusions

This case report highlights the challenges and potential strategies in managing PTCL-NOS, especially when traditional and novel therapies such as CHOP regimen, brentuximab, or romidepsin fail. *TET2* gene mutation can be found in some cases of PTCL-NOS. It is implicated in epigenetic regulation via DNA methylation. Thus, hypomethylating agents such as azacytidine have been theorized to be effective in addressing the *TET2* mutation. Our patient's successful treatment with azacytidine, selected based on a *TET2* mutation, emphasizes the importance of genetic profiling in guiding therapy for refractory PTCL-NOS. Future research could focus on further investigating azacytidine’s efficacy in this patient population and exploring the broader utility of epigenetic therapies in PTCL.
